# Supplementary material for: Metal tolerance capacity and antioxidant responses of new Salix spp. clones in a combined Cd-Pb polluted system
Source: PeerJ. 2022 Dec 16;10:e14521. doi: 10.7717/peerj.14521 (PMC9762249; doi:10.7717/peerj.14521)
Supplement: Supplemental Information 2 [file peerj-10-14521-s002.docx]

| Treatment group | Cd (μM) | Pb (μM) |
| --- | --- | --- |
| CK | 0 | 0 |
| LCdLPb | 15 | 250 |
| HCdLPb | 30 | 250 |
| LCdHPb | 15 | 500 |
| HCdHPb | 30 | 500 |
